# Improving ungated steady‐state cardiac perfusion using transition bands

**DOI:** 10.1002/mrm.30467

**Published:** 2025-02-18

**Authors:** Jason K. Mendes, Johnathan V. Le, Andrew E. Arai, Ravi Ranjan, Edward V. R. DiBella, Ganesh Adluru

**Affiliations:** ^1^ Utah Center for Advanced Imaging Research, Department of Radiology and Imaging Sciences University of Utah Salt Lake City Utah USA; ^2^ Department of Biomedical Engineering University of Utah Salt Lake City Utah USA; ^3^ Division of Cardiovascular Medicine University of Utah Salt Lake City Utah USA

**Keywords:** arterial input function, cardiac perfusion, ungated perfusion

## Abstract

**Purpose:**

Although gated first‐pass contrast‐enhanced sequences are the clinical standard for cardiovascular MR perfusion, some patient conditions necessitate using ungated steady‐state sequences. However, through‐plane cardiac motion and blood flow into the left ventricle can disrupt the magnetization steady state of the tissue, and perfusion quantification based on a steady‐state assumption will contain errors. The tissue magnetization steady‐state disruption can be eliminated with a proposed sequence modification that simultaneously excites transition bands with no change in the sequence resolution or timing parameters.

**Theory and Methods:**

The proposed sequence modification simultaneously excites two transition bands adjacent to the imaged region. The transition bands experience the same consistent excitation history as the imaged slices. Thus, any tissue that moves into an imaged slice location from a transition band location will not disrupt the magnetization steady state. Gradient dephasing and radiofrequency spoiling are used to null the transition band signal so that it does not contribute to the reconstructed images. Transition bands were added to a two‐dimensional ungated steady‐state radial FLASH (fast low‐angle shot) sequence with simultaneous multiband imaging on a PRISMA 3T MRI scanner. Phantom, normal canine, and human subject data are presented.

**Results:**

Transition bands reduce the amount of tissue magnetization disruption to the steady state without adding artifacts to the imaged slices. Myocardial blood flow maps from a selected normal canine study and a normal human subject show good uniformity and consistency to literature values for all slices. Perfusion estimates with the proposed method also demonstrate good consistency with saturation‐recovery methods commonly used for myocardial perfusion imaging.

**Conclusion:**

We have demonstrated that the proposed transition bands can reduce quantification errors resulting from blood flow into the left ventricle and through‐plane cardiac and respiratory motion. There is no loss of image‐acquisition efficiency, and temporal resolution is unchanged with this technique.

## 1 INTRODUCTION

Quantitative cardiac MR perfusion is a successful noninvasive imaging technique to detect ischemic heart disease.^1^, ^2^, ^3^, ^4^, ^5^, ^6^, ^7^ The dominant clinical option for cardiac MR perfusion is gated first‐pass contrast‐enhanced perfusion imaging.^8^, ^9^, ^10^, ^11^ However, in some patients with cardiac arrhythmia^12^ or inadequate electrocardiogram signals,^13^ gated images are misclassified into the wrong cardiac phase or corrupted by motion artifacts.^14^, ^15^ This is an important consideration, as the worldwide prevalence of atrial fibrillation has increased by 33% in the last 20 years, with incidence rates as high as 17% in those older than 80 years.^16^ Alternative gating methods can help with inadequate electrocardiogram signals,^17^, ^18^ but in patients with cardiac arrhythmia, ungated steady‐state sequences may be useful.^19^, ^20^, ^21^, ^22^, ^23^, ^24^, ^25^, ^26^ Ungated steady‐state sequences use a consistent application of radiofrequency (RF) excitations, either steady‐state free precession or spoiled gradient echo, to acquire data across varying cardiac phases without additional magnetization preparation.^27^, ^28^ The varying cardiac phase during image acquisition allows images to be retrospectively reconstructed at any cardiac phase but also creates new hurdles to accurate perfusion quantification.

To quantify first‐pass contrast‐enhanced perfusion with ungated steady‐state sequences, the tissue magnetization is converted to gadolinium concentrations for use in a pharmacokinetic model.^29^, ^30^, ^31^ This conversion is modeled on the assumption that the tissue magnetization in an imaged slice has reached a steady state. For static tissue, steady‐state magnetization can be maintained across all the image slices by interleaving the excitation of slices (i.e., each slice receives a single excitation before moving on to the next slice). If tissue in a magnetization steady state moves from one imaged slice to another, the disruption to the magnetization steady state is relatively small. However, the magnetization steady‐state disruption becomes significant when tissue moves into an imaged slice from outside the imaged region.

In this case, the tissue moving into the imaged slice is unexcited or in an unknown excited state, and multiple RF excitations must be applied to return the imaged slice to a steady magnetization state. For example, even for the relatively high excitation flip angle of 30°, Sharif et al. report that it still takes about 24 RF excitations before the myocardium reaches 90% of the steady‐state contrast‐to‐noise ratio between precontrast and peak contrast signals.^20^ The longer blood relaxation time requires even more RF excitations to reach a suitable magnetization steady state. Consequently, blood flowing into an imaged region may not reach magnetization steady state until after it has crossed several imaged slices. The near‐constant cardiac and respiratory motion and blood flow into the left ventricle can prevent the edge slices from ever reaching magnetization steady state. Therefore, the conversion of tissue magnetization to gadolinium concentrations will have some errors related to the motion‐induced disruption of the magnetization steady state. This is particularly noticeable when quantifying the arterial input function (AIF), as blood tends to have higher velocities, travels larger distances, and moves in a more chaotic trajectory than myocardium.

The most straightforward and least efficient solution is to discard one or more slices from the edges of the imaged region. To maintain the spatial coverage of the imaged region, the total number of imaged slices would need to be increased by the number of discarded slices at the cost of a longer acquisition time. Instead of discarding the edge slices, large excitation flip angles can reduce the disruption to the magnetization steady state.^20^ One pitfall of this proposal is that large flip angles are not optimal to discern between normal and diseased myocardial tissue. Another proposed method uses nonselective RF excitations interleaved with slice‐selective excitations.^32^ This method can reduce the disruption to the magnetization steady state but at the cost of reduced image‐acquisition efficiency and decreased temporal resolution.

**FIGURE 1:**
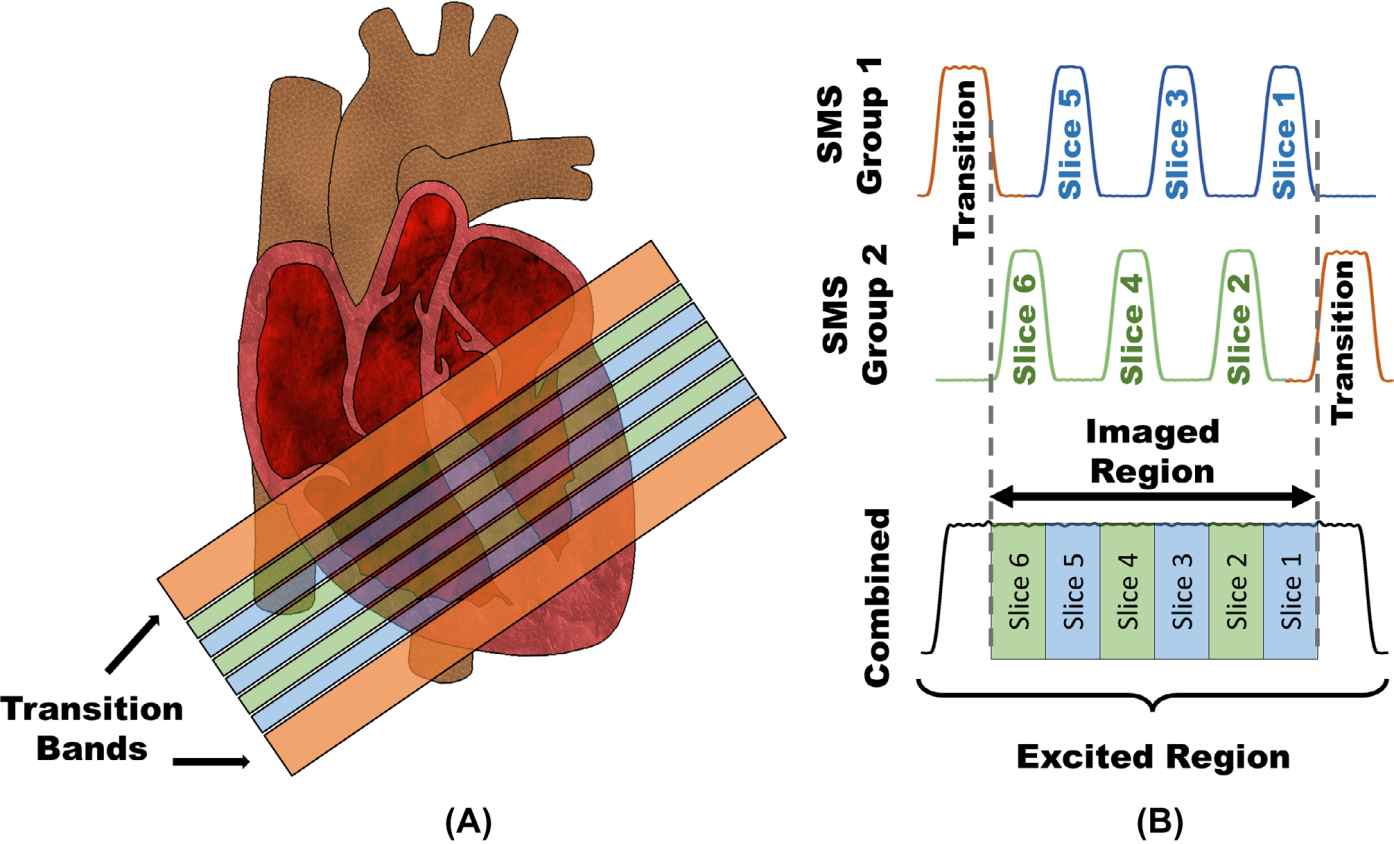
(A) The proposed sequence excites a transition band on each end of an imaged region. (B) To consistently excite the transition bands and imaged slices, each transition band is excited with only one of the slice groups. All tissue in the excited region receives consistent excitation, but only tissue in the imaged region contributes to the reconstructed images. SMS, simultaneous multislice.

This work proposes a sequence modification to ungated steady‐state perfusion, which can eliminate the disruption to the magnetization steady state due to through‐plane tissue motion. Two transition bands adjacent to the imaged region (Figure 1) are excited simultaneously and consistently with the imaged slices. Tissue moving through a transition band will be driven to steady‐state magnetization consistent with the imaged slices before reaching the imaged region. Consequently, there is no disruption to the magnetization steady state when this tissue moves into an imaged slice. Although the transition bands have the same RF excitation history as the imaged slices, the transition‐band magnetization is immediately nulled such that it does not contribute to the reconstructed images. As a result, only minor changes are necessary for image reconstruction, with no change to the imaging protocol or acquisition timing. We demonstrate the use of transition bands with a two‐dimensional (2D) radial fast low‐angle shot (FLASH) ungated steady‐state sequence to quantify perfusion in canine and human studies.

## 2 THEORY

This work uses radial simultaneous multislice imaging, which combines the RF excitation waveforms of $N_{s}$ slices (equal to the multiband factor) to excite a group of slices at the same time. To prevent an increase in acquisition time, the transition bands are also excited simultaneously with the imaged slices by further addition of the transition‐band RF waveform to the composite RF waveform of the slice group. To accommodate multiple slice groups and reduce peak B_1_ during excitation, each transition band is excited independently with one of the slice groups (Figure 1B). Each transition band will experience the same number of excitation pulses per unit time as any slice group. For this work, six slices (two slice groups, $N_{s}=3$) were acquired with one transition band excited with each slice group. Additional slice groups may be added with only the first and last slice groups, including a transition band. In the case of a single imaged slice, both transition bands and the imaged slice can be excited at the same time.^33^ However, to accommodate simultaneous multislice imaging and to reduce peak B_1_ during excitation, each transition band is excited independently with one of the slice groups (Figure 1B).

**FIGURE 2:**
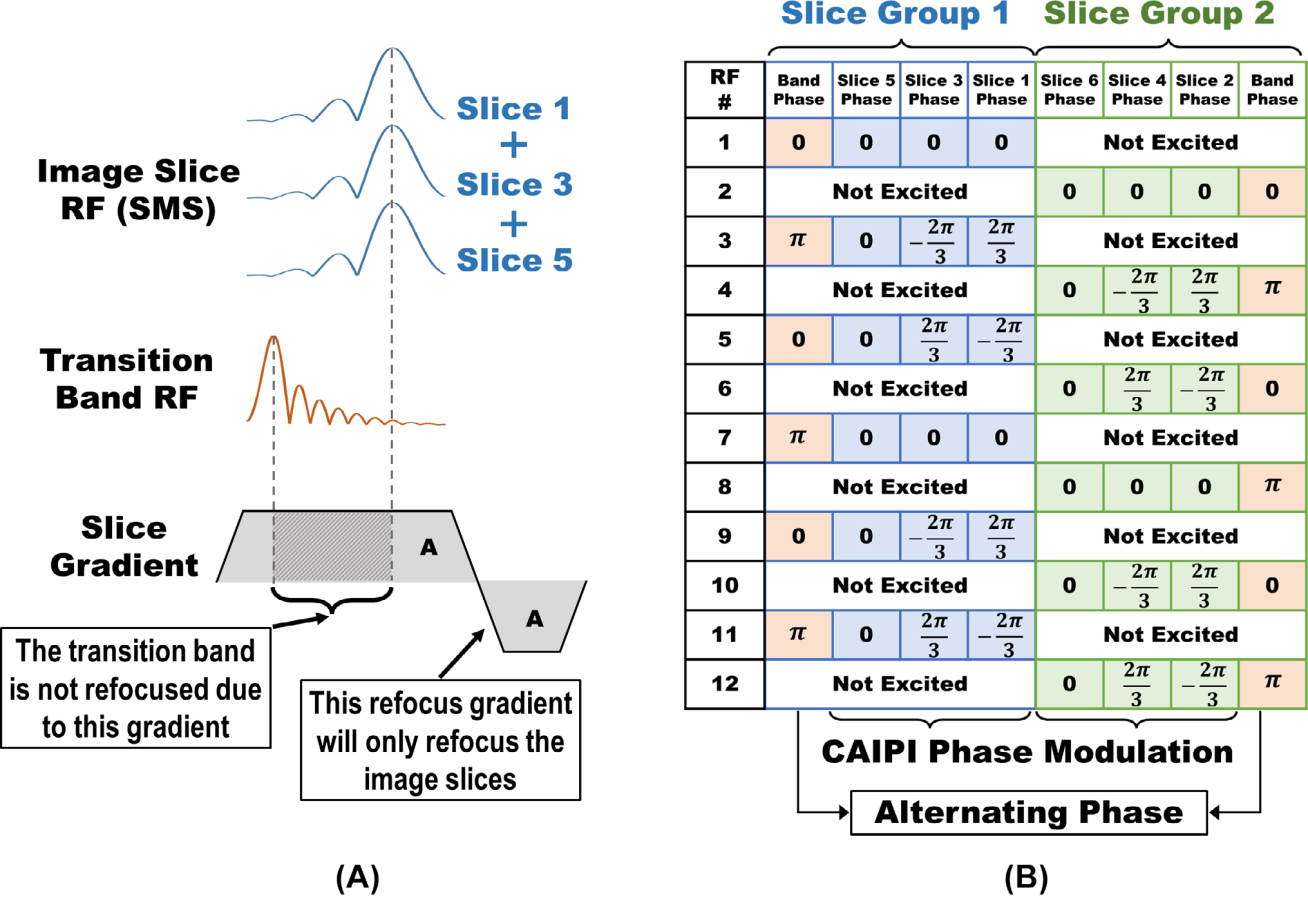
(A,B) Transition band signal nulling through gradient dephasing (A) and radiofrequency (RF) spoiling (B). The transition‐band RF waveform has an early asymmetric time to the B_1_ peak, whereas the image‐slice RF waveforms have a late asymmetric time to B_1_ peak, as shown in (A). The shaded slice‐select gradient in (A) between the two B_1_ peak locations is not included in the slice refocus gradient and results in a linear phase induced across the transition band, and consequential gradient dephasing of the signal. The transition‐band signal can also be nulled using a modified phase‐cycling scheme, as shown in (B). The alternating phase of each transition band excitation (*orange table entries*) will result in signal nulling if the appropriate number of excitations is used in the image reconstructions (in this case, each slice group should use a multiple of six excitations). CAIPI, controlled aliasing in parallel imaging.

All RF waveforms in the composite pulse are applied simultaneously with a single slice‐select gradient (Figure 2A). The image slice thickness and the bandwidth of the image‐slice RF waveform determine the magnitude of the slice‐select gradient. Therefore, the simultaneously excited transition band thickness can only be adjusted by changing the bandwidth of the transition RF waveform. Ideally, the transition band should be as large as possible with a sharp slice profile. Unfortunately, hardware B_1_ limitations and patient specific absorption rate (SAR) limits place an upper limit on the maximum bandwidth of the transition RF pulse and, consequently, the transition band thickness. This tradeoff will be considered more in Section 5.

We use gradient dephasing and RF spoiling to null any signal from the transition band (Figure 2B). Any slice‐select gradient applied after the B_1_ peak of an RF waveform will need to be refocused. If not refocused, a linear phase and associated signal dephasing will occur across the excited slice. However, the necessary slice refocus gradient can only fully refocus the linear phase associated with a single B_1_ peak location. If the image‐slice RF waveforms and the transition‐band RF waveform have different B_1_ peak locations, then a slice‐refocus gradient can be tailored only to refocus the image slices. To prevent an increase in scan time, the transition‐band RF waveform must be applied at the same time and for the same duration as the image‐slice RF waveform. To achieve an offset in the B_1_ peak locations, the image‐slice RF waveforms have a late asymmetry (B_1_ peak occurs after half the duration of the RF waveform), and the transition‐band RF waveform has an early asymmetry (B_1_ peak occurs before half the duration of the RF waveform). A slice‐refocus gradient designed to refocus image slices excited by late B_1_ peak RF waveforms will not refocus the transition band excited with an early B_1_‐peak RF waveform (Figure 2A).

For the radially acquired image slices, the RF phase modulations are set using controlled aliasing in parallel imaging (CAIPI).^34^, ^35^, ^36^ Adopting the notation introduced by Yutzy et al.,^36^ the sampled k‐space data of a slice group from the *m*th excitation of a CAIPI phase modulated sequence is

(1) $$S\left(m\right)=\sum_{\alpha =1}^{N_{S}}\phi \left(\alpha ,m\right)S_{\alpha }\left(m\right)$$

where $S_{\alpha }\left(m\right)$ is the k‐space data of each individual slice at index α; ϕ(α,m) is the RF waveform phase modulation of the individual slice; and $N_{s}$ is the number of simultaneously excited slices. The corresponding image reconstruction of a slice at index b after *M* excitations is

(2) $$\rho_{b}=R\left(\sum_{m=1}^{M}\phi^{*}\left(b,m\right)S\left(m\right)\right)$$

where R is a general reconstruction operator that includes gridding and a Fourier transform, and $\phi^{*}\left(b,m\right)$ is the complex conjugate of the RF waveform phase modulation of the individual slice. The slice‐group k‐space data in Eq. (1) can be extended to include a transition band as follows:

(3) $$\tilde{S}\left(m\right)=S\left(m\right)+\varphi \left(m\right)T\left(m\right)$$

where T(m) is the k‐space data of the transition band from the $m^{\mathrm{th}}$ excitation, and φ(m) is the phase modulation of the transition band RF waveform. The reconstructed slices in Eq. (2) will now also include an error term, $\varepsilon_{b}$, derived from any residual transition band signal not nulled by gradient dephasing. The reconstructed image slices with the residual transition band signal are then

(4) $${\tilde{\rho }}_{b}=\rho_{b}+\varepsilon_{b}$$

where

(5) $$\varepsilon_{b}=R\left(\sum_{m=1}^{M}\phi^{*}\left(b,m\right)\varphi \left(m\right)T\left(m\right)\right)$$

In this work, we use a common CAIPI phase modulation for the image‐slice RF waveforms (Figure 2B) as follows:

(6) $$\phi \left(\alpha ,m\right)=e^{-i\frac{2\pi \left(\alpha -1\right)m}{N_{s}}}$$

If the number of excitations is a multiple of double the multiband factor ($M=2N_{s}\cdot N$), then the single summation in Eq. (5) can be split into a double summation as follows:

(7) $$\varepsilon_{b}=R\left(\sum_{n=1}^{N}\sum_{l=1}^{2N_{s}}e^{i\frac{2\pi \left(b-1\right)l}{N_{s}}}\varphi \left(m\right)T\left(m\right)\right)$$

where

(8) $$m=2N_{s}\left(n-1\right)+l$$

Finally, if the transition band signal is slowly varying over the small number of $2N_{s}$ excitation pulses

(9) $$T\left(m\right)\approx T\left(2N_{s}n\right),$$

then the error term can be closely approximated as

(10) $$\varepsilon_{b}\approx R\left(\sum_{n=1}^{N}T\left(2N_{s}n\right)\sum_{l=1}^{2N_{s}}e^{i\frac{2\pi \left(b-1\right)l}{N_{s}}}\varphi \left(m\right)\right)$$

Consider the case with no transition‐band phase modulation such that φ(m)=1. The error contribution from the transition slice would be

(11) $$\varepsilon_{b}\approx \left\{\begin{array}{ll} R\left(2N_{s}\sum_{n=1}^{N}T\left(2N_{s}n\right)\right) & b=1\\ 0 & b\neq 1 \end{array}\right.$$

because

(12) $$\sum_{l=1}^{2N_{s}}e^{i\frac{2\pi \left(b-1\right)l}{N_{s}}}=\left\{\begin{array}{ll} 2N_{s} & b=1\\ 0 & b\neq 1 \end{array}\right.$$

**FIGURE 3:**
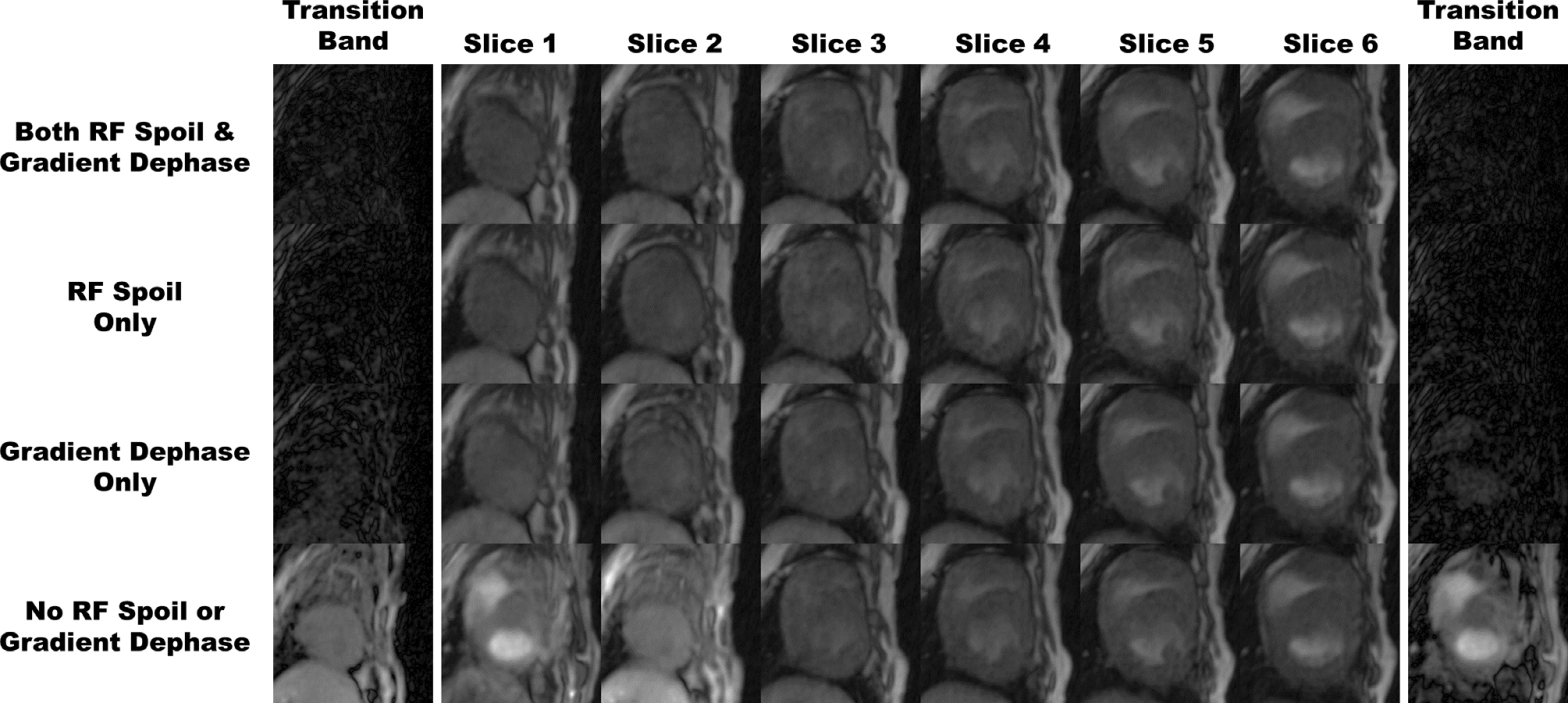
Canine (HR =92bpm) residual transition‐band signal after various nulling methods. The effects of only using radiofrequency (RF) spoiling (*second row*), only using gradient dephasing (*third row*), or combining both nulling methods (*top row*) are shown. The residual transition‐band signal without any nulling is shown in the bottom row.

We note that with no RF phase modulation, the transition‐band signal only contributes an error term to the first slice in each slice group (artifact in the first two slices of Figure 3). If we add a phase modulation to the transition‐band RF waveform as follows:

(13) $$\varphi \left(m\right)=\left\{\begin{array}{ll} e^{-i\pi m} & \mathrm{Odd}\;N_{s}\\ e^{-i\pi \left(m+\text{floor}\left(\frac{m-1}{N_{s}}\right)\right)} & \text{Even}\;N_{s} \end{array}\right.$$

then $\varepsilon_{b}=0$ for all reconstructed slices, as

(14) $$\sum_{l=1}^{2N_{s}}e^{i\frac{2\pi \left(b-1\right)l}{N_{s}}}e^{-i\pi l}=\sum_{l=1}^{2N_{s}}e^{i\frac{2\pi \left(b-1\right)l}{N_{s}}}e^{-i\pi \left(l+\text{floor}\left(\frac{l-1}{N_{s}}\right)\right)}=0$$

Consequently, RF phase modulation can null the transition band signal if we adopt the RF waveform phase modulations in Eqs. (6) and (13), and the number of excitations is a multiple of twice the multiband factor ($M=2N_{s}\cdot N$).

## 3 METHODS

All studies were performed on a Prisma 3T MRI scanner (Siemens Healthcare, Erlangen, Germany) with animal studies approved by the Institutional Animal Care and Use Committee. Human data were acquired with written consent following institutional review board policies. The base ungated steady‐state sequence was a continuous 2D radial FLASH with an echo time (TE)/repetition time (TR) of 0.75/2.1ms, 1.8×1.8×7mm acquisition resolution (260×260mm field of view), six short‐axis slices acquired in two groups (multiband factor of 3), golden‐angle radial CAIPI^36^ with a $12^{{}^{\circ}}$ flip angle. The image‐slice groups were excited using late asymmetric filtered sinc pulses with three side lobes (refocus gradient area is 26.6% of the slice gradient) and RF phase modulations described by Eq. (6).

The sequence was modified to add simultaneously excited transition bands, as shown in Figure 1. The transition bands were excited using an early asymmetric filtered sinc pulse with five side lobes (refocus gradient area would need to be 86.7% of the slice gradient) and RF phase modulations described by Eq. (13). The image slices and the transition band RF waveforms were 900μs in duration, with a peak B_1_ time difference of 540μs. The transition‐band RF waveform bandwidth was 2.49 times larger than the image‐slice RF waveforms, corresponding to a transition band thickness of 17.4mm and an image‐slice thickness of 7mm. To facilitate the conversion of signal intensity to $T_{1}$, a set of proton density–weighted images was also acquired with parameters like these, except the flip angle was reduced to $2^{{}^{\circ}}$.

To evaluate the accuracy of blood flow quantification using the proposed sequence, we also acquired ungated saturation‐recovery data sets for comparison. The ungated saturation‐recovery sequence was a 2D radial FLASH sequence with a TE/TR of 1.1/2.6ms, 1.8×1.8×7mm acquisition resolution (260×260mm field of view), three short‐axis slices acquired in one group (multiband factor of 3), golden‐angle radial CAIPI^36^ with a $8^{{}^{\circ}}$ flip angle. The single‐slice group also had a saturation recovery time of 10ms with 30 rays acquired every saturation pulse.

Ungated steady‐state images were reconstructed from 12 rays per frame using a spatiotemporal constrained reconstruction with patch‐based low‐rank regularization and an ADMM optimization algorithm. Because the number of cardiac phases across cardiac cycles can vary due to heart‐rate changes or arrhythmia, the number of cardiac phases was fixed for all cardiac cycles using a ray‐grouping algorithm based on the cardiac signal acquired from a 0.5−2.2Hz bandpass filtering.^28^, ^37^ The ungated saturation‐recovery images were reconstructed from 30 rays per frame using a pixel‐tracking spatiotemporal constrained reconstruction with patch‐based low‐rank regularization. Image‐based self‐gating was performed to bin images into systole and diastole using a left‐ventricular blood pool mask to extract the cardiac signal.^27^

The reconstructed images were registered using the parametric total variation (pTV) registration toolbox with a group‐wise nuclear metric and an isotropic total variation regularization of displacements.^38^ Afterward, the images were further registered using a model‐based registration procedure. Model‐based images were generated by fitting signal curves to the two‐compartment model.^30^, ^31^ These model‐based images were then used as reference motion‐free images to perform deformable registration, where the pTV‐registered images were further deformed to the motion‐free model‐based images.^39^, ^40^

A dictionary was generated using Bloch equations (the protocol parameters described previously) and slice profiles of the simultaneous multislice excitations and transition bands. Signal‐intensity curves were normalized by the proton density signal and converted to $T_{1}$ relaxation times using the generated dictionary and a pattern‐recognition algorithm like Marty et al.^41^ The relaxation times were then converted to gadolinium concentrations using

(15) $$\frac{1}{T_{1}}=\frac{1}{T_{1,0}}+r\left[\mathrm{Gd}\right]$$

where *r* and *[Gd]* are the relaxivity and concentration of the gadolinium contrast agent,^42^ and $T_{1,0}$ is the baseline relaxation time before gadolinium contrast injection. For perfusion studies, regions of interest in the left‐ventricular blood pool were drawn on each slice, and the corresponding signal‐intensity curves were used to generate the AIFs. The myocardium was segmented using the American Heart Association 16‐segment model.^43^ A slice‐averaged AIF, the myocardium gadolinium concentration curves, and a two‐compartment model^30^, ^31^ were used to generate pixel‐wise myocardial blood flow maps. The final pixel‐wise myocardial blood flow maps were then median‐filtered. The two‐compartment model used was

(16) $$C_{\mathrm{tis}}\left(t\right)=C_{\mathrm{bld}}\left(t-∆t\right)⨂K^{\text{trans}}e^{-k_{\mathrm{ep}}t}+V_{b}C_{\mathrm{bld}}\left(t-∆t\right)$$

where $C_{\mathrm{tis}}\left(t\right)$ and $C_{\mathrm{bld}}\left(t\right)$ are the tissue and blood [Gd] curves; $K^{\text{trans}}$ is the kinetic rate constant of contrast agent flow from the vasculature into the extravascular, extracellular myocardium; $k^{\mathrm{ep}}$ is the kinetic rate constant of contrast agent flow from the extravascular, extracellular myocardium into the vasculature; $V_{b}$ is the component of tissue enhancement due to myocardial vasculature; and ∆t is the time delay between blood and tissue enhancement. In this work, we report $K^{\text{trans}}$ as an index of myocardial blood flow. Myocardial blood flow quantification was calculated from the systolic image series of the ungated steady‐state and ungated saturation‐recovery sequences after retrospective self‐gating.

Flow pump phantom experiments used a 1‐cm‐diameter tubing perpendicular to the image‐slice orientation. A simple motor controller (Pololu Robotics & Electronics) and custom software regulated power to a DC brushless water pump. Flow velocities were initially calibrated using MR phase‐contrast imaging and were verified again with this experiment. Gadoteridol (ProHance; Bracco Diagnostics) was added to tap water to mimic possible $T_{1}$ times of blood at peak ($T_{1}=63\;\mathrm{ms}$) and postinjection ($T_{1}=234\;\mathrm{ms}$) of a gadolinium contrast agent.^44^ The $T_{1}$ relaxation times were measured with the transition‐band ungated steady‐state sequence described previously. Two transition‐band thicknesses were tested (7mm and 17.4mm) along with no transition bands. A region drawn inside the tubing for each of the six slices was used to calculate $T_{1}$ across multiple flow velocities, and $T_{1}$ error was quantified using a reference $T_{1}$ measured with a clinical modified Look‐Locker inversion recovery (MOLLI) sequence. The MOLLI sequence used a 5(3)3 acquisition artificially triggered with a 60−bpm simulated electrocardiogram, the water pump turned off, 1.4×1.4×7mm acquisition resolution, and a centric gradient echo–based readout. Simulated data were generated in the same manner as the dictionary described previously, except after each excitation pulse, a fraction of the longitudinal magnetization is replaced with fully recovered magnetization. The fraction of replaced magnetization was determined by the slice thickness, TR, and the simulated flow velocity (assuming uniform flow).

Results from four canine subjects (HR=92,96,97, and 111bpm) and a human subject (HR=56bpm) are included in this study. One canine subject had an infarct in the inferior wall of the left‐ventricular myocardium due to alcohol ablation treatment targeting the circumflex artery, affecting the left‐ventricular free wall. The canine studies used two 0.06mmol/kg injections of Gadoteridol (ProHance), whereas the human study used two 0.075mmol/kg injections of Gadobutrol (Gadovist; Bayer Healthcare Pharmaceuticals). All studies described were free breathing. No dual‐bolus^45^, ^46^, ^47^ or dual‐sequence^48^, ^49^, ^50^ methods were used to generate the AIF or tissue signal curves for the proposed ungated steady‐state sequence. All studies used an injection rate of 4 mL/s with at least 15 min between subsequent contrast injections.

## 4 RESULTS

Figure 3 demonstrates the effectiveness of gradient dephasing and RF spoiling to null the signal of the transition bands for a normal canine subject (HR=92bpm). The transition band signal is well nulled with either gradient dephasing or RF spoiling alone. However, the gradient dephasing alone still has some noticeable residual transition band signal (third row in Figure 3), especially in the blood pools. The combination of both gradient dephasing and RF spoiling yields the best transition band nulling. We note some residual signal in the chest wall; however, this signal would not interfere with accurate myocardial blood‐flow quantification. The residual transition band signal, if any, is visualized in the first slice of each slice group (Slice 1 and Slice 2 in Figure 3).

**FIGURE 4:**
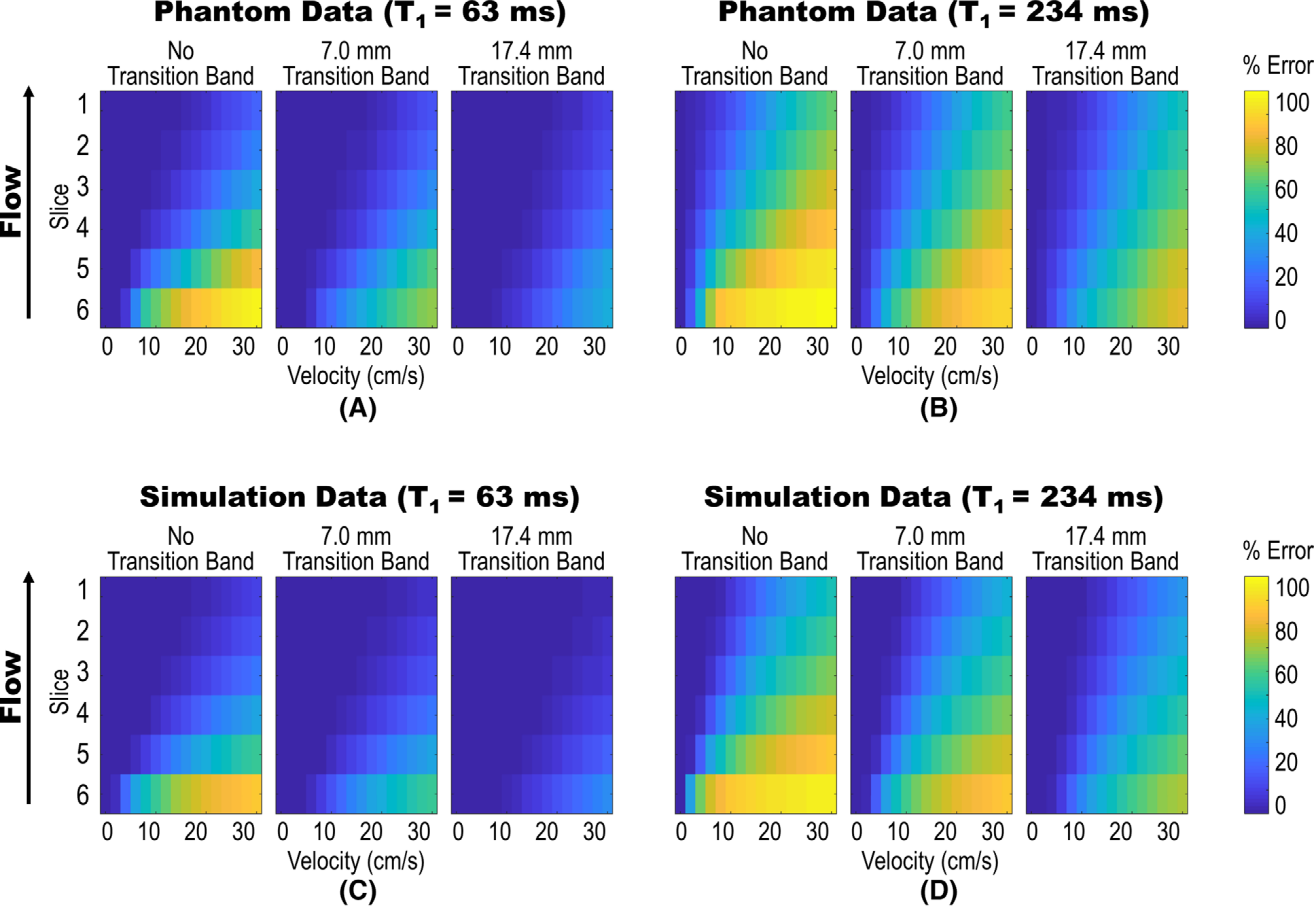
The transition‐band thickness influences how well magnetization steady state can be maintained in the presence of inflow. The title above each set of error maps indicates which configuration was used (no transition bands, 7−mm transition bands, and 17.4−mm transition bands). Quantified $T_{1}$ error maps generated from MR phantom data acquired with a flow pump are shown in the top row (A,B), with matching Bloch simulations shown in the bottom row (C,D). Two different $T_{1}$ times were investigated to represent blood $T_{1}$ during peak contrast uptake (*left side*, $T_{1}=63\;\mathrm{ms}$) and postcontrast injection (*right side*, $T_{1}=234\;\mathrm{ms}$). The flow direction was from the bottom (Slice 6) to the top (Slice 1), with velocities varied up to 30cm/s. The intensity of the error maps represents the error in quantifying $T_{1}$, with higher (*yellow*) intensities indicating more error.

The error in quantifying $T_{1}$ due to inflow is illustrated in Figure 4. MR phantom data (Figure 4A,B) and simulation results (Figure 4C,D) are shown for an ungated 2D radial FLASH sequence with and without transition bands. Generally, the amount of error increased with flow velocity and was less pronounced the further slices were along the flow direction (less error in Slice 1 vs. Slice 6 in Figure 4). Acquired phantom data (top row in Figure 4) matched well with simulated data (bottom row in Figure 4). Both the 7‐mmand 17.4−mm transition bands reduced the quantification error, with the larger transition band offering larger improvements. Figure S1 demonstrates signal‐evolution simulations using a sinusoidal motion model with HR=120bpm and a maximum displacement of one slice thickness for the ungated steady‐state sequence with and without transition bands. These simulations demonstrate an edge‐slice average $T_{1}$ motion error of <1% versus 37% with and without the transition band, which suggests all slices would be suitable for blood‐flow quantification, even with significant motion, when using the proposed method (see Supporting Information S1 for additional details).

**FIGURE 5:**
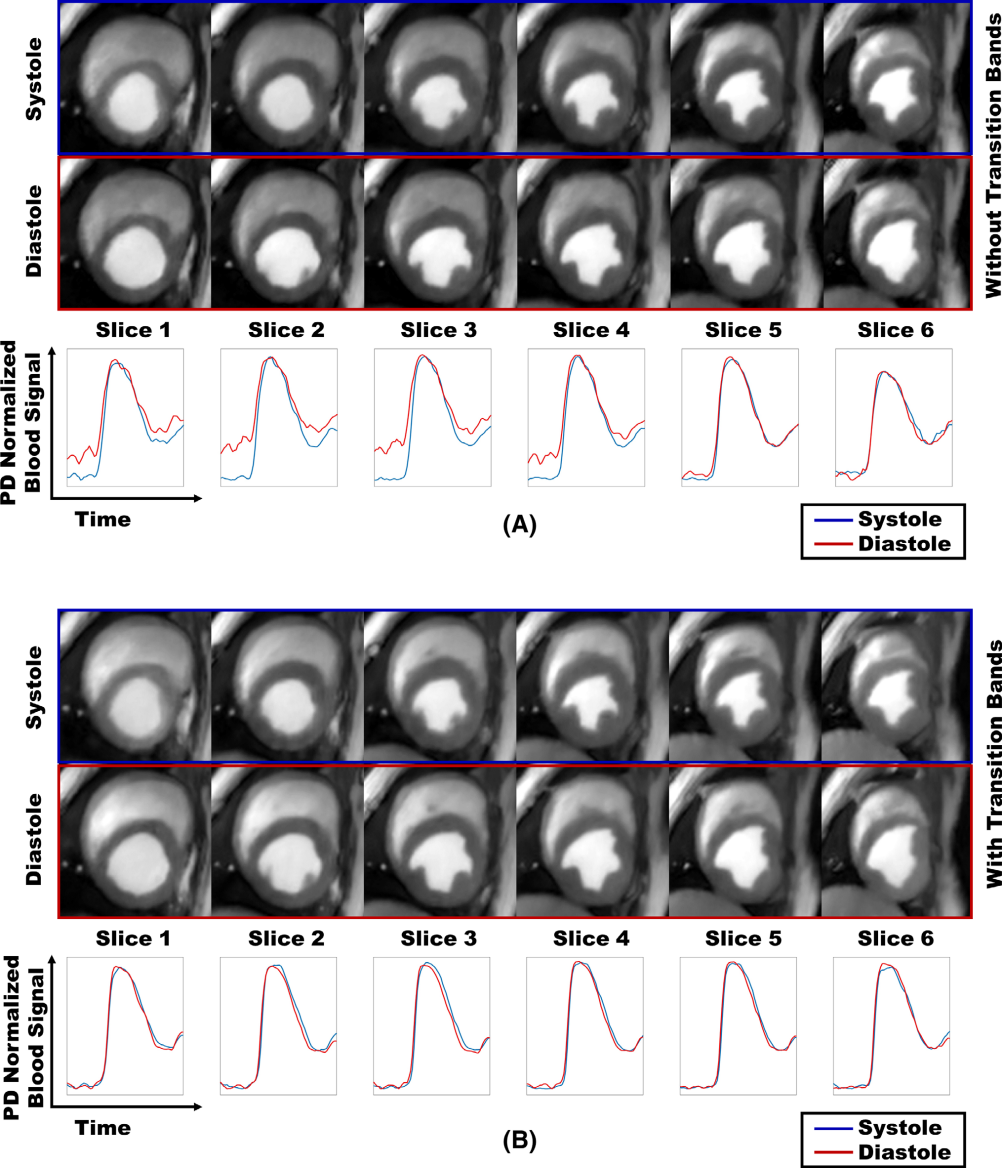
(A,B) Canine study (HR =96bpm) with an ungated steady‐state radial two‐dimensional FLASH (fast low‐angle shot) sequence without (A) and with (B) transition bands. Because the sequence is ungated, images can be reconstructed from any part of the cardiac cycle with both systolic and diastolic reconstructions shown. Regions were drawn in the left‐ventricle blood pool for each slice and cardiac phase, with the signal intensities (normalized by the proton densities) plotted below each set of images. PD, proton density.

Blood‐signal errors due to blood flow into the left ventricle for a normal canine subject (HR=96bpm) are shown in Figure 5. Because the data are ungated, images can be reconstructed at any cardiac phase, with systolic and diastolic phases reconstructed in this study (shown near peak AIF uptake in the blood pool in Figure 5). Qualitatively, the image sets with and without the transition bands show no remarkable differences. However, the blood signal in the left ventricle without the transition bands shows significant differences between systolic and diastolic values, as illustrated by the red and blue lines in Figure 5A. These blood‐signal differences are more pronounced toward the base of the heart and less noticeable during peak contrast uptake. When the transition bands are used (Figure 5B), the blood pool signals from the left ventricle in systole and diastole show better agreement.

**FIGURE 6:**
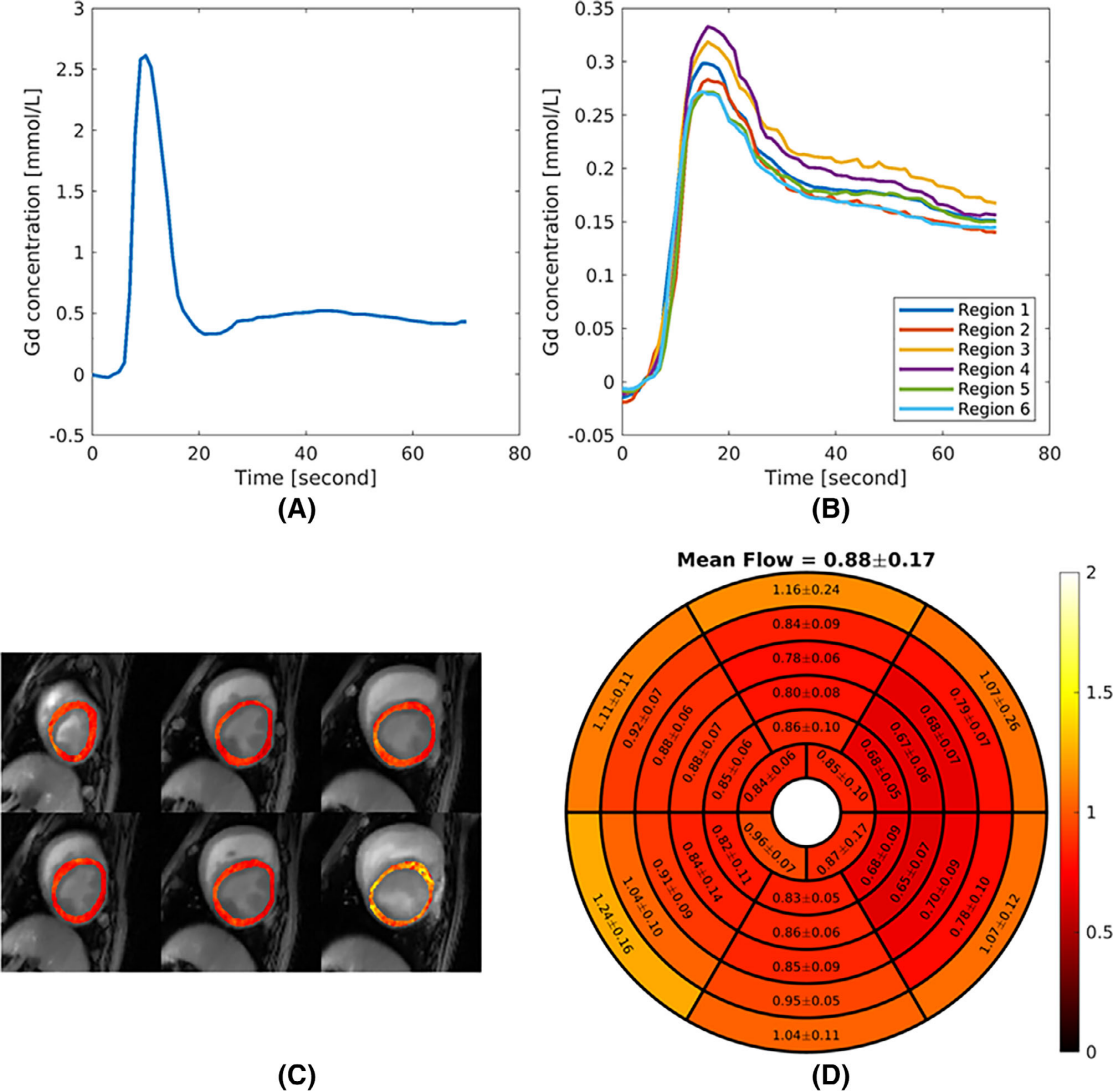
Resting myocardial blood‐flow quantification results (reported as K^trans^) for a normal canine subject (HR =111bpm) using transition bands. (A–C) Shown are the arterial input function (A), averaged tissue curves for one midventricular slice (B), pixel‐wise perfusion maps for all six slices (C), and the bullseye perfusion summary of each myocardial territory based on the American Heart Association 16‐segment model. Gd, gadolinium.

**FIGURE 7:**
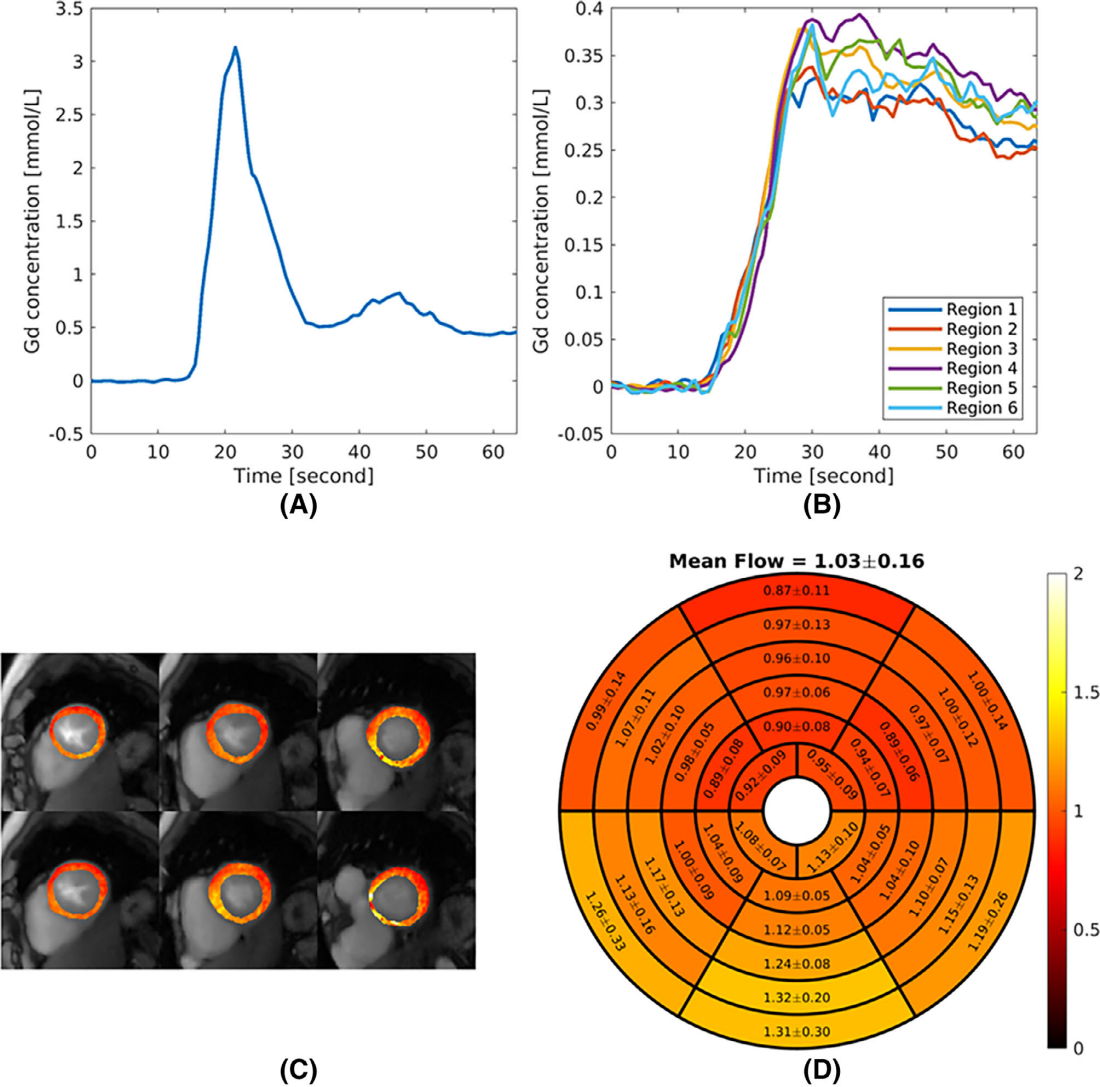
Resting myocardial blood‐flow quantification results (reported as K^trans^)for a normal human subject (HR =56bpm) using transition bands. (A–C) Shown are the arterial input function (A), averaged tissue curves for one midventricular slice (B), pixel‐wise perfusion maps for all six slices (C), and the bullseye perfusion summary of each myocardial territory based on the American Heart Association 16‐segment model. Gd, gadolinium.

The results for ungated CMR perfusion quantification of a normal canine subject (HR=111bpm) and a normal human subject (HR=56bpm) are shown in Figures 6 and 7, respectively. The AIF and the tissue curves for one midventricular slice are shown, and the remaining slices also show consistent gadolinium concentration curves. Pixel‐wise perfusion maps from both studies demonstrate good uniformity throughout all the acquired slices (Figures 6C and 7C). The mean myocardial K^trans^  for the normal canine and normal human subjects were $0.88\pm 0.17\;\min^{-1}$ and $1.03\pm 0.16\;\min^{-1}$ (Figures 6D and 7D). These estimates are similar to previously published literature values ranging from $0.52\;\min^{-1}$ to 1.2 min^−1^.^30^ The edge basal slices in both subjects show similar uniformity and flow values to those of the midventricular slices. Movies of the reconstructed images for this canine and human subject can be found in Videos S1 and S2, respectively.

**FIGURE 8:**
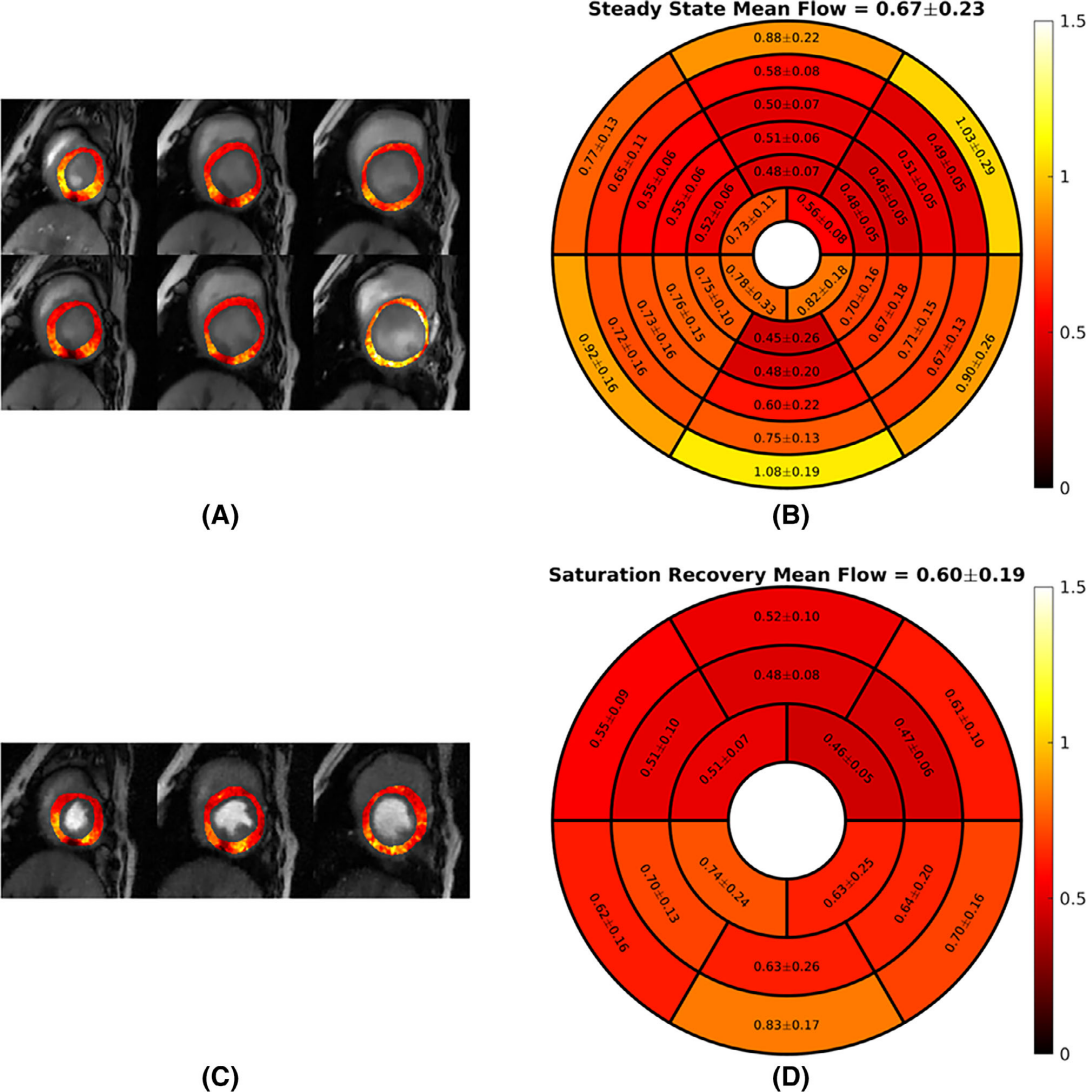
Resting myocardial blood flow quantification results (reported as K^trans^) for a canine subject with an infarcted inferior wall of the left‐ventricular myocardium (HR =97bpm) due to alcohol ablation treatment targeting the circumflex artery, affecting the left‐ventricular free wall. (A,B) Pixel‐wise perfusion maps (A) and bullseye perfusion summary (B) of each myocardial territory for the proposed ungated steady‐state sequence with transition bands. (C,D) Pixel‐wise perfusion maps (C) and bullseye perfusion summary (D) of each myocardial territory for the ungated saturation recovery sequence.

Figure 8 demonstrates ungated steady state and saturation‐recovery perfusion quantification for a canine subject (HR=97bpm) with an infarcted inferior wall in the left‐ventricular myocardium. The proposed ungated steady‐state sequence ($0.67\pm 0.23\;\min^{-1}$) demonstrates similar flow estimates to a saturation‐recovery sequence ($0.60\pm 0.19\;\min^{-1}$) commonly used for clinical myocardial perfusion imaging, suggesting that the proposed method can produce accurate myocardial blood‐flow maps. Movies of the reconstructed images for the proposed ungated steady‐state sequence and the saturation‐recovery sequence for this canine subject can be found in Videos S3 and S4, respectively.

## 5 DISCUSSION

Although gated first‐pass contrast‐enhanced perfusion is the dominant clinical CMR technique for disease evaluation, some patient conditions necessitate using ungated methods. Accurate quantification relies on a tissue magnetization steady state for ungated steady‐state CMR perfusion. Through‐plane cardiac and respiratory motion and blood flow into the left ventricle disrupt this magnetization steady state and result in quantification errors. Applying transition bands to ungated steady‐state sequences can reduce this disruption to the magnetization steady state.

The proposed ungated steady‐state sequence excited three slices simultaneously by combining three 900−μs RF waveforms. An additional 900−μs waveform was used for the transition bands, resulting in a composite of four total RF waveforms. The total duration of the composite RF excitation remains at 900μs with no change to any imaging parameters, including TE, TR, all gradients, sampling, and total scan time. Consequently, only existing gradients and RF phase modulation of the transition band are leveraged to null the transition band signal. We also note that the CAIPI phase modulation of the image slices contributes to some inherent signal nulling of the transition band. Specifically, for the CAIPI phase modulation in Eq. (6), the residual transition‐band signal will only manifest in the first slice of each slice group according to Eq. (11) (see the bottom row of Figure 3).

The use of transition bands only has two sequence and reconstruction considerations. First, the number of rays used to reconstruct a slice must be twice the multiband factor. This varies from the base ungated steady‐state sequence using CAIPI, which only requires the number of rays to be a multiple of the multiband factor. Second, there is an increase in the total RF power applied with the addition of the transition‐band RF waveform, which could limit the types of transition bands used due to SAR or the maximum RF amplifier voltage.

Because the slice‐select gradient is unchanged with the addition of the transition bands, the thickness and slice profile can only be varied by changing the shape or bandwidth of the transition‐band RF waveform. Generally, we would like to use the thickest transition band with the sharpest profile possible. Practically, sharper profiles and thicker transition bands require a higher peak voltage and increased SAR. Because the transition band is applied simultaneously with the other image‐slice RF waveforms, the maximum voltage of the RF amplifier may limit the voltage peak of the added transition‐band RF waveform. For the Prisma 3T MRI scanner used in this work, we were never limited by peak RF amplifier voltage. However, an upper limit of the transition band thickness was encountered when SAR limits were reached. If a thicker transition band is necessary, then the total excitation duration would need to be increased, and the scan time would no longer remain the same as the original ungated steady‐state sequence. Because we desired to keep all imaging timing the same, we kept the 900−μs pulse duration unchanged and found an asymmetric filtered sinc pulse with five side lobes was possible, given the required SAR limitations. This transition‐band RF waveform had a bandwidth that was 2.49 times he bandwidth of the imaging‐slice RF waveform. Consequently, for an image slice thickness of 7mm, the corresponding transition band thickness would be 17.4mm.

A study by Kim et al. and others measured mitral annulus and mitral leaflet blood velocities during atrial filling as 27cm/s and 35cm/s, respectively.^51^ Furthermore, studies by Shechter et al. and Jagsi et al. report peak myocardium velocities due to cardiac and respiratory motion as 5cm/s and 0.8cm/s, respectively.^52^, ^53^ We investigated the effectiveness of a 17.4−mm‐thick transition band using a flow phantom with velocities up to about 30cm/s. We also compared the 17.4−mm transition band to a 7−mm transition band, simulating the effect of discarding a single 7−mm image slice from each end of the imaged region. Although both transition band thicknesses reduced $T_{1}$ quantification errors and analogously to gadolinium concentration errors in perfusion studies, the thicker band performed better (Figure 4). All studies in this work were performed with free breathing and at rest. Even for deep breathing, Jagsi et al. report maximum myocardium velocities of about 3cm/s, which is still relatively small compared with the velocity of blood flowing into the left ventricle. For the short‐axis slice orientation used in this work, respiratory myocardium motion tends to manifest as more in‐plane motion (which does not disrupt the magnetization steady state) than through‐plane (which disrupts the magnetization steady state).

One way to visualize the disruption to the magnetization steady state is to observe the blood signal in the left ventricle across the cardiac cycle (Figure 5). If the systolic and diastolic blood signals do not match, the blood flow into the left ventricle disrupts the magnetization steady state. In Figure 4, these disruptions are minimal in the most apical slices and during systole. However, recent studies have verified that more accurate AIFs are obtained from the base of the heart,^54^ and imaging during systole is more challenging. To allow the AIF to be quantified from a basal slice during diastole, transition bands will help reduce the magnetization steady‐state disruption. We also observe that the short blood $T_{1}$ during peak contrast‐agent concentration drives the blood signal to steady state quickly, even in the presence of blood inflow. Consequently, most magnetization steady‐state disruption will occur before and after the peak contrast‐agent concentration (as seen in the proton density–normalized signal intensity curves in Figure 5A). Quantification errors due to cardiac motion and blood flow into the left ventricle are most prominent in the edge basal slices, as these slices contain the most recent inflow during image acquisition. In both subjects, the edge basal slices show similar uniformity and flow values as the midventricular slices, suggesting that the transition bands can facilitate the quantification of the edge slices. The perfusion estimates obtained in the canine and human normal subjects (Figures 6 and 7) are similar to previously published literature values.^30^ In addition, perfusion estimate comparisons between the proposed ungated steady‐state sequence and an ungated saturation‐recovery sequence (Figure 8) suggest good quantification accuracy with more standard clinical sequences for myocardial perfusion imaging. However, rigorous analysis with a larger sample size and statistical methods is required to verify the efficacy of the new transition band method, which will be the focus of future work.

Vasodilation studies may require additional demands on the transition bands. Because one canine study had a heart rate of 111bpm, we suspect the increased heart rate of vasodilation in human studies would not require additional consideration. Motion simulations using a heart rate of 120bpm and a maximum displacement of one slice thickness also suggest that our method can handle the increased heart rate of vasodilation with stress imaging. However, the current transition band thickness of 17.4mm may not be large enough with the expected increase of blood velocity into the left ventricle during vasodilation. This would only manifest in the AIF quantification, particularly in the edge basal slices (see Figure 5 for an example of AIF changes between basal and apical slices). Increasing the TR or reducing slice‐profile sharpness may be necessary to allow a thicker transition band. We plan to investigate this more fully in future vasodilation studies.

It is also important to note that there is a physiological upper limit to transition‐band thickness. For left‐ventricular imaging, the transition band must only cover the entire volume of the left atrium to be completely effective. We also note that for a 7−mm image‐slice thickness, the 17.4−mm transition band is equivalent to discarding 2.5 slices on each edge of the imaging region. Consequently, for higher vasodilation blood flow velocities into the left ventricle, the 17.4−mm transition band may not be adequate for the edge basal slices but may be adequate to quantify the AIF in the more apical slices. This work was done on a Siemens Prisma 3T scanner. However, the proposed sequence modification can also be readily extended to 1.5T scanners. The reconstruction parameters can be optimized for the decreased signal from the lower field strength.

If an ungated steady‐state sequence is necessary to quantify CMR perfusion, this work has demonstrated that transition bands are an easy and effective sequence modification to reduce perfusion errors. This is especially true when quantifying the diastolic AIF in basal slices, eliminating the need for a dual bolus or a dual‐sequence protocol. Although transition bands do increase the overall SAR of the imaging sequence, they do not require any changes to the gradients or timing of ungated steady‐state sequences.

## Supporting information


**FIGURE S1.** Signal evolution simulations ($T_{1}=234\;\mathrm{ms}$) of the ungated steady‐state sequence and with and without transition bands using a no‐motion model (A,D) and a sinusoidal motion model with HR=120bpm and a maximum displacement of one slice thickness (B,E). The $T_{1}$ motion error for $T_{1}$ ranging from 1ms to 1500ms was also examined for the ungated steady‐state sequence with (C) and without (F) transition bands. Only Slices 1–3 were examined due to identical behavior with Slices 4–6.

**VIDEO S1.** Movie demonstrating the proposed ungated steady‐state perfusion reconstructionsused to generate the myocardial blood flow maps of the normal canine subject shown in Figure 6.

**VIDEO S2.** Movie demonstrating the proposed ungated steady‐state perfusion reconstructions used to generate the myocardial blood flow maps of the healthy human subject shown in Figure 7.

**VIDEO S3.** Movie demonstrating the proposed ungated steady‐state perfusion reconstructions used to generate the myocardial blood flow maps of the canine subject with an infarcted inferior wall of the left ventricular myocardium shown in Figure 8.

**VIDEO S4.** Movie demonstrating the ungated saturation‐recovery perfusion reconstructions used to generate the myocardial blood flow maps of the canine subject with an infarcted inferior wall of the left ventricular myocardium shown in Figure 8.

## Data Availability

We will make the deidentified raw data for the human subject, as well as reconstruction, quantification, and simulation code, publicly available. Files can be downloaded at https://github.com/gadluru.
