# Supplementary material for: Improving ungated steady‐state cardiac perfusion using transition bands
Source: Magn Reson Med. 2025 Feb 18;94(1):199–214. doi: 10.1002/mrm.30467 (PMC12021336; doi:10.1002/mrm.30467)
Supplement: Supplementary file 1 — FIGURE S1. Signal evolution simulations (T1=234ms) of the ungated steady‐state sequence and with and without transition bands using a no‐motion model (A,D) and a sinusoidal motion model with HR=120bpm and a maximum displacement of one slice thickness (B,E). The T1 motion error for T1 ranging from 1ms to 1500ms was also examined for the ungated steady‐state sequence with (C) and without (F) transition bands. Only Slices 1–3 were examined due to identical behavior with Slices 4–6. [file MRM-94-199-s003.docx]

Supporting Information Figure S1 illustrates signal evolution simulations ($T_{1}=234 ms$) of the ungated steady state sequence with and without the transition bands, using no motion model and using a sinusoidal motion model with $HR=120 bpm$ and a maximum displacement of one slice thickness (only slices 1-3 have been illustrated as slices 4-6 exhibit identical behavior). Each point along the curves shown in (a), (b), (d), and (e) represents a single excitation with 3000 total excitations simulated and a time resolution of 2*TR ($TR=2.4 ms$) between each point due to the interleaved acquisition. With the transition band technique, the edge slices (slices 1 and 6 in Supporting Information Figure S1) experience the same crosstalk interference as the center slices, resulting in consistent signal evolution curves between all the excited slices. Even under high heart rates like those seen during stress imaging, the transition band technique ensures that moving tissue experiences an excitation history like non-moving tissue, resulting in significant signal variation reductions compared to the same sequence with no transition slices. The edge slices are most affected by motion, resulting in an average $T_{1}$ estimation error of $37\%$ for slices 1 and 6 (see Supporting Information Figure S1f) and $11\%$ for slices 2 and 5 after reaching steady state with $T_{1}=1500 ms$. For slices 3 and 4, motion causes an average $T_{1}$ estimation error of $<1\%,$ suggesting only these slices would be suitable for MBF quantification without the transition band technique under these motion conditions. However, edge slices only experience an average $T_{1}$ estimation error of $<1\%$ (see Supporting Information Figure S1c) using the transition band technique after reaching steady state with $T_{1}=1500 ms,$ suggesting that all slices would be suitable for MBF quantification even with significant motion.


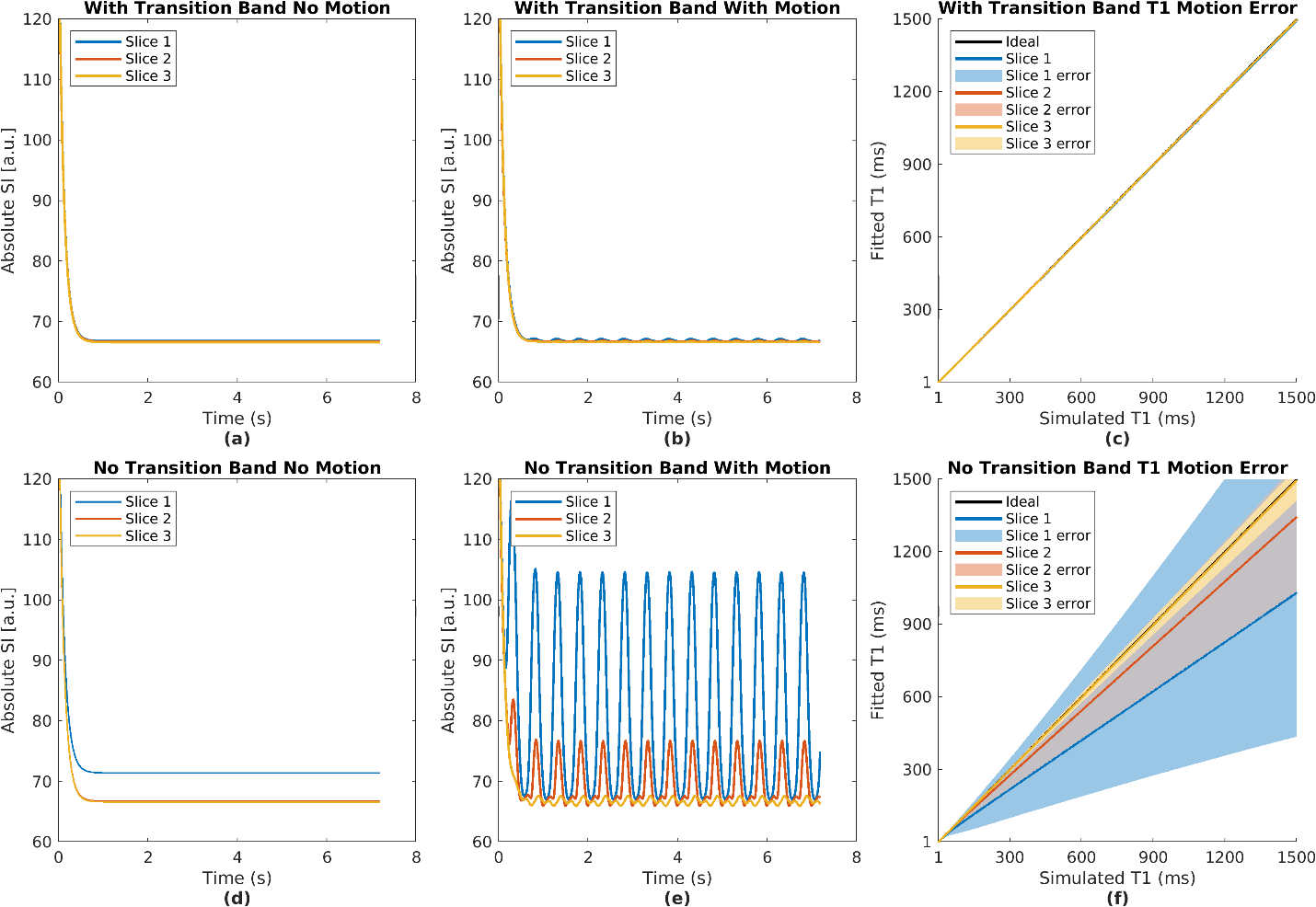


**Supporting Information Figure S1.** Signal evolution simulations ($T_{1}=234 ms$) of the ungated steady state sequence and with and without transition bands using **(a, d)** no motion model and a **(b, e)** sinusoidal motion model with $HR=120 bpm$ and a maximum displacement of one slice thickness. The $T_{1}$ motion error for $T_{1}$ ranging from $1 ms$ to $1500 ms$ was also examined for the ungated steady state sequence **(c)** with and **(f)** without transition bands. Only slices 1-3 were examined due to identical behavior with slices 4-6.
